# Enzyme Catalyzed Copolymerization of Lignosulfonates for Hydrophobic Coatings

**DOI:** 10.3389/fbioe.2021.697310

**Published:** 2021-07-14

**Authors:** Sebastian A. Mayr, Nikolaus Schwaiger, Hedda K. Weber, Janez Kovač, Georg M. Guebitz, Gibson S. Nyanhongo

**Affiliations:** ^1^Institute of Environmental Biotechnology, University of Natural Resources and Life Sciences, Tulln, Austria; ^2^Sappi Papier Holding GmbH, Gratkorn, Austria; ^3^Department of Surface Engineering, Jozef Stefan Institute, Ljubljana, Slovenia; ^4^Austrian Centre of Industrial Biotechnology, Tulln, Austria

**Keywords:** lignosulfonate, hydrophobicity, fluorophenol, coupling, coating, laccase, modification

## Abstract

Enzymatic polymerization of lignin can generate a variety of value-added products concomitantly replacing fossil-based resources. In line with this approach, a laccase from the thermophilic fungus *Myceliophthora thermophila* (MtL) was used to couple a hydrophobicity enhancing fluorophenol (FP) molecule, namely 4-[4-(trifluoromethyl)phenoxy]phenol (4,4-F3MPP), as a model substrate onto lignosulfonate (LS). During the coupling reaction changes in fluorescence, phenol content, viscosity and molecular weight (size exclusion chromatography; SEC) were monitored. The effects of enzymatic coupling of FP onto LS on hydrophobicity were investigated by the means of water contact angle (WCA) measurement and determination of swelling capacity. Full polymerization of LS resulting in the production of water-insoluble polymers was achieved at a pH of 7 and 33°C. Incorporation of 2% (w/v) of FP led to an increase in WCA by 59.2% while the swelling capacity showed a decrease by 216.8%. Further, Fourier transform infrared spectroscopy (FTIR) and X-ray photoelectron spectroscopy (XPS) analysis indicated successful covalent coupling of the FP molecule onto LS by an emerging peak at 1,320 cm^–1^ in the FTIR spectrum and the evidence of Fluor in the XPS spectrum. This study shows the ability of laccase to mediate the tailoring of LS properties to produce functional polymers.

## Introduction

**FIGURE 1:**
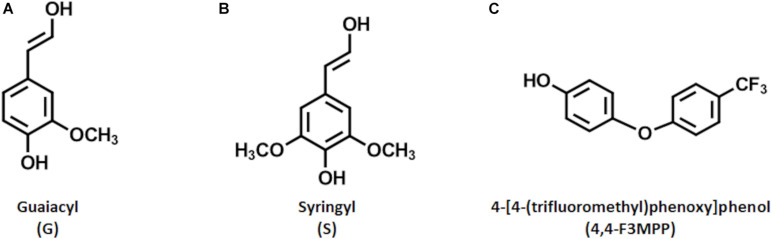
Structures of the relevant reactive molecules. Shown are the monolignol units G **(A)** and S **(B)** of lignin (the H unit is not shown due to its low abundance in wood biomass). Further, the Fluorophenol molecule, 4-[4-(trifluoromethyl)phenoxy]phenol (4,4-F3MPP), used for the reaction is shown **(C)**.

As society nowadays becomes more and more aware of the problems linked to climate change and environmental pollution, huge efforts are made to replace fossil-based materials (Hoffert et al., 2002; Ragauskas et al., 2006). Promising alternatives are found in renewable natural and sustainable resources like lignin (Kumar et al., 2020). Lignin represents the second most abundant natural biopolymer and, together with cellulose and hemicellulose, forms the matrix of biomass (Ponnusamy et al., 2019). While cellulose and hemicellulose have traditionally been used to produce valuable products, like high-quality paper, lignin has always been regarded as a low-value by-product and is mainly burned for energy generation. This is due to the complex and inhomogeneous structure thereof. Lignin is composed mainly of three repetitive building blocks, represented by *p*-hydroxyphenyl (H), syringyl (S), and guaiacyl (G). These so called monolignols differ in the number of methoxy groups present in their structure. The G unit has one methoxy group on position three of the aromatic ring, while the S unit has two of them at positions three and five (Figures 1A,B). The distribution of these monolignols defines the reactivity of the respective lignin (Vanholme et al., 2010).

However, lignin, representing the biggest pool of natural aromatics, is meanwhile thought to have the potential to also be used for high-value applications. Nevertheless, only about 2% of the annually produced lignin is used commercially these days for example as an additive in binders (Calvo-Flores and Dobado, 2010; Schutyser et al., 2018).

Depending on the type of pulping process used for isolation, products with different properties, known as technical lignins, are generated. The most common forms of these processes are sulfite, kraft, organosolv, and soda pulping. In the sulfite pulping process lignosulfonates (LS) are produced, resulting in a water-soluble form of lignin through the introduction of sulfonate groups during the pulping process (Constant et al., 2016; Li and Takkellapati, 2018). A possible application thereof was recently demonstrated by our group, where surface coatings were synthesized through laccase mediated polymerization of LS (Ortner et al., 2018).

**FIGURE 2:**
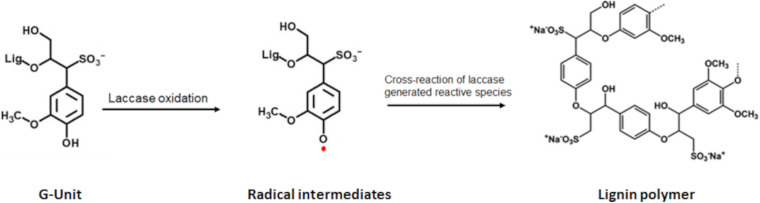
Scheme of laccase catalyzed oxidation of lignin. Exemplary shown for the G-unit. Upon laccase oxidation radicals are formed which can cross react with each other thereby forming lignin polymers.

Laccases (EC 1.10.3.2, *p*-diphenol:dioxygen oxidoreductase) occur in plants, fungi, bacteria, and insects. They are predominant in fungi, where they assist in the biodegradation of lignin (Santhanam et al., 2011; Mate and Alcalde, 2017). Laccases are multicopper proteins that oxidize phenols and aliphatic or aromatic amines to radicals while reducing molecular oxygen to water (Riva, 2006; Giardina et al., 2010; Mate and Alcalde, 2017). In the case of LS, laccases interact mainly with the phenolic residues. Upon oxidation of the hydroxyl moiety of the monolignols, phenoxy radicals are formed, which can cross-react among each other or with foreign molecules (Figure 2; Morozova et al., 2007; Solomon et al., 2008; Madhavi and Lele, 2009; Bassanini et al., 2021).

As stated before, a novel combination of processes was developed in our group, that enables extensive laccase mediated polymerization of LS when a steady external oxygen supply is granted resulting in an increased dispersibility (Nugroho Prasetyo et al., 2010; Ortner et al., 2015, 2018; Huber et al., 2016). Recently, the best conditions for this laccase mediated polymerization process of LS to achieve big polymers in a short time were investigated (Weiss et al., 2020). The thus generated insoluble polymers were promising to be applied in surface coatings. Presenting a potential alternative to fossil-based binders used e.g., in paper coating formulations where styrene-butadiene or styrene-acrylate latex are used. The findings were, that the produced LS polymers performed nearly as good as the actually used latex-based binders (Ortner et al., 2018). However, a possible way to improve the performance would be an increase in hydrophobicity, since penetration of the LS containing coatings into hydrophilic cellulose would then be reduced which is beneficial. In general, a higher hydrophobicity of LS would be beneficial for both this and some other potential applications (Calvo-Flores and Dobado, 2010; Cusola et al., 2013; Ortner et al., 2015). In this regard, previous studies have demonstrated the ability of laccase to mediate the coupling of different fluorophenol molecules (FP) onto lignocellulose fibers and lignin model substrates, resulting in an increased hydrophobicity. The best results were found when 4-[4-(trifluoromethyl)phenoxy]phenol (4,4-F3MPP) (Figure 1C) was used as a substrate (Kudanga et al., 2010; Acero et al., 2014; Wu et al., 2016; Song et al., 2019).

Unlike LS, kraft lignin (KL) is insoluble in water and may be considered as a less complex alternative to obtain hydrophobic lignin coatings, without the need for coupling of foreign molecules at all. Unfortunately, it was shown that e.g., pine Indulin AT (a commercially available KL) cannot be polymerized to the same extent as LS, making it unsuitable to be used in such coatings (Ortner et al., 2015, 2018).

However, in all these before-mentioned studies, FP was grafted either on lignocellulose fibers or lignin model substrates. Whereas, herein this respective FP molecule was coupled directly onto isolated LS. The enzyme used was isolated from the thermophilic fungus *Myceliophthora thermophila* which has potential for several biotechnical applications (Singh, 2016). The effects of this enzymatically catalyzed coupling of the hydrophobicity enhancing model FP compound during an ongoing polymerization of LS was investigated under various reaction conditions. The FP molecule was chosen as a model substrate since it allows for simple detection of successful coupling events onto LS polymers using measurements like Fourier transform infrared spectroscopy (FTIR) and X-ray photoelectron spectroscopy (XPS).

## Materials and Methods

### Materials

All used chemicals were purchased from Sigma-Aldrich (Steinheim, Germany) or Merck (Darmstadt, Germany) and were of analytical grade. *Myceliophthora thermophila* laccase (MtL) was obtained from Novozymes (Novozyme 51003, Bagsveard, Denmark). The herein used LS originates from used liquor, generated during the sulfite wood pulping process and was kindly provided by Sappi.

### Laccase Activity Assay

Laccase activity was determined by monitoring the oxidation of 2,2′-azino-bis(3-ethylbenzothiazoline-6-sulfonic acid) diammonium salt (ABTS) to its cation radical. The resulting reaction product was measured at 420 nm by using a plate reader (Tecan, Infinite M200, Switzerland). For dilution of the enzyme, a 50 mM phosphate buffer with a pH of 7 was used. For the reaction, 170 μL of the diluted enzyme was mixed with 50 μL of a 10 mM ABTS solution and measured immediately. The blank consisted of 170 μL of phosphate buffer mixed with 50 μL of the ABTS solution. All measurements were done in triplicates. The activity was expressed in Unit (defined as the amount of enzyme necessary to convert 1 μmol substrate per 1 min). For the reaction, the volumetric activity in U/mL was calculated.

### Enzymatic Polymerization of Lignosulfonate

Laccase mediated polymerization was carried out in a 100 mL glass bottle containing 80 mL of a 10% (w/v) LS solution. The solution was set to the respective pH using a 2 N NaOH and aerated at 30 mL/min. When a constant level of aeration and the respective reaction temperature was reached, the reaction was started by the addition of 10 U/mL of MtL. For incorporation of FP, the respective concentration was added 30 min after the reaction was started. The blank sample contains FP but no enzyme. The respective control samples contain the enzyme but no FP. In order to monitor the progress of the reaction, samples were drawn in regular intervals for further analyses.

### Determination of Phenol Content

The content of phenolic groups in LS was determined using the Folin–Ciocalteu (FC) method. The FC-reagent reacts with phenol groups and forms a blue phosphotungstic–phosphomolybdenum complex that can be quantified by UV/VIS spectroscopy (Blainski et al., 2013). The reaction mixture containing 20 μL LS sample and 60 μL FC-reagent was filled up to a final volume of 680 μL with MQ-water and was incubated for 5 min at 21°C. Thereafter, 200 μL sodium carbonate 20% (w/v) and 120 μL MQ-water were added. The samples were incubated for 2 h at 21°C and 800 rpm. After incubation, 200 μL of the treated sample were transferred into a 96-well plate and the absorbance was measured at 760 nm with a plate reader (Tecan Infinite M200). The measured blank contained MQ-water instead of LS and was otherwise treated like the samples. The phenol concentration in the LS samples was calculated by using vanillin as standard in concentrations ranging from 0.05 to 1 mg/mL. All samples were measured in triplicates.

### Fluorescence Measurement

The fluorescence intensity during enzymatic polymerization of LS was measured as described by Nugroho Prasetyo et al. (2010) with some modifications. One hundred microliter of lignin samples were mixed with 120 μL of an aqueous solution of 2-methoxyethanol (2:1 v/v) in a 96 well plate. The sample was excited at 355 nm and the emitted light was measured at 400 nm. The fluorescence measurement was done on a plate reader (Tecan Infinite M200). All samples were measured in triplicates.

### Viscosity Measurement

The change in viscosity was followed throughout the reaction by applying 700 μL of the respective sample onto the Rheometer (MCR 302, Anton Paar, Austria) equipped with a measuring system consisting of a cone plate with a diameter of 50 mm and an angle of 1° (CP50-1). The viscosity measurement was done for 10 s with measuring points made every second at a constant temperature of 20°C and a constant shear rate of 200 s^–1^. Data analysis was done with the Anton Paar software RheoCompass 1.24. All samples were measured in duplicates.

### Size-Exclusion Chromatography

The molecular weights of the samples were determined using size exclusion chromatography (SEC). A liquid chromatography system equipped with a quaternary/binary pump, autosampler 1260 series, a DAD, a RI-detector system (Agilent Technologies 1260 Infinity) and a MALLS HELEOS DAWN II detector (Wyatt Technology, Santa Barbara, CA, United States) was used. The columns used consisted of a precolumn PL aqua gel-OH MIXED guard (PL1149-1840, 8 μm, 7.5 mm × 50 mm, Agilent, Palo Alto, CA, United States) and a separation column PL aqua gel-OH MIXED H (PL1549-5800, 8 μm, 4.6 mm × 250 mm, Agilent, Palo Alto, CA, United States) with a range from 6 to 10,000 kDa. The mobile phase contained 50 mM NaNO_3_ with 200 mg/L NaN_3_. The sample injection volume was 100 μL and the total runtime for one sample was 90 min. The lignins were diluted with the mobile phase to a concentration of 1 mg/mL before loading onto the column. For normalization, band broadening and alignment of the MALLS detector a BSA standard was used. The software used for data acquisition and analysis was the ASTRA 7 software from Wyatt Technologies.

### Water Contact Angle

For determination of the water contact angle, 10 mL of the samples were poured into Petri dishes and dried at room temperature to produce flat films. The films were left at room temperature for 1 day, until when completely dry. The hydrophobicity was measured using the contact angle technique. A drop shape analyzer device (DSA100, Krüss, Germany) was used for the measurements. The contact angle between a drop of water and the surface of the sample is determined. Therefore, a picture is taken at the moment of deposition allowing determination of the contact angle by the DSA 4.0 Software by Krüss. All samples were measured in triplicates.

### Swelling Capacity

The hydrophobicity was further investigated by determination of the amount of water absorbed by the samples. Before the measurement, the samples were washed in water and 70% (v/v) ethanol, respectively followed by drying in the 70°C oven. The samples were put into an empty beaker and weighed, followed by addition of MQ-water. After 1 h of soaking, the excess water was carefully removed and the samples were weighed again. To determine the swelling capacity the amount of water uptake by the sample was calculated. To get the swelling capacity in % the calculated mass for the absorbed water (m_water_) is divided by the mass of the weighed in dry sample (m_dryweight_) and multiplied with 100 (1). The amount of water was calculated by subtracting the mass of the dry sample (m_dryweight_) from the mass of the wet sample (m_wet_) after soaking for 1 h in water (2).

(1) $$S\left[\%\right]=\left(m_{water}/m_{dryweight}\right)*100$$

(2) $$m_{water}=m_{wet}-m_{dryweight}$$

### Fourier Transform Infrared Spectroscopy

The washed and dried samples were milled and a small amount of the powder was directly applied to the ATR-FTIR device (Spectrum 100, PerkinElmer). The spectrum was recorded in a range from 4,000 to 600 cm^–1^ with 30 scans and a resolution of 4 cm^–1^. The spectra were baseline corrected and normalized over the fingerprint region of LS (between 1,070 and 930 cm^–1^). All samples were measured in triplicates and the results were averaged to obtain the spectrum for each sample.

### X-Ray Photoelectron Spectroscopy

The coupling of FP onto LS was determined using XPS. Analyses were carried out on the PHI-TFA XPS spectrometer produced by Physical Electronics Inc. (Chanhassen, MN, United States) and equipped with a monochromatic Al-source. The analyzed area was 0.4 mm in diameter and the analyzed depth was about 3–5 nm. Low energy electron neutralizer was used to avoid sample charging. During data processing, the spectra were aligned by setting the C 1s peak at 284.8 eV, characteristic for C–C/C–H bonds. Quantification of surface composition was performed from XPS peak intensities taking into account relative sensitivity factors provided by the instrument manufacturer (Moulder et al., 1993). Every sample was analyzed at two different places and the average composition was calculated.

## Results and Discussion

In order to find out the best-suited conditions for the successful enzymatic coupling of FP molecules onto LS a prescreening was done under acidic (pH 5), neutral (pH 7), and alkaline pH (pH 8). It is well known that amongst other parameters the pH of a reaction does influence enzyme activity, not least due to the impact on the redox potentials of both the substrates (LS and FP) and the enzyme (MtL) in this particular reaction (Xu, 1997; Tadesse et al., 2008).

The results for the modified samples (LS+MtL, addition of FP after 30 min of ongoing reaction) were compared to control samples (LS + MtL, without addition of FP), a blank sample (LS without MtL but with addition of FP after 30 min) and a POLY+FP sample (LS + MtL, addition of FP after the reaction was stopped after 150 min, followed by stirring only for another 30 min). The delayed addition of FP is thought to increase the likelihood of an immediate reaction between FP and the already formed LS reactive species, but also to grant a certain degree of polymerization, which is also needed for better dispersibility. Therefore, the FP was added after 30 min to the ongoing reaction.

**FIGURE 3:**
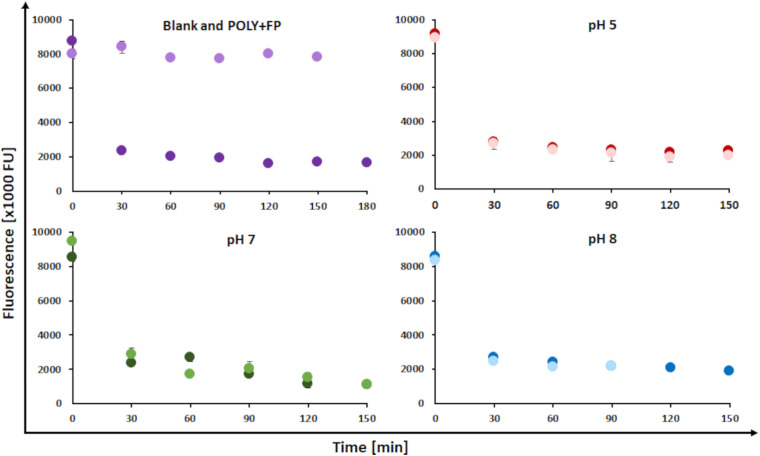
Changes in fluorescence during laccase catalyzed coupling of FP onto LS at a temperature of 23°C, addition of 1% (w/v) FP and at different pH conditions. **(Upper left)** Blank sample (LS without enzyme, FP added after 30 min; light purple dots) and POLY + FP sample (LS + MtL, FP added in the end of the reaction after 150 min; dark purple dots). **(Upper right)** Control sample (LS + MtL, without FP added; dark red dots) and modified sample (LS + MtL, addition of FP after 30 min; light red dots) at pH 5. **(Lower left)** Control sample (darkt green dots) and modified sample (light green dots) at pH 7. **(Lower right)** Control sample (dark blue dots) and modified sample (light blue dots) at pH 8.

The decrease in fluorescence was used as a parameter to monitor the reactions as previously described (Albinsson et al., 1999; Ortner et al., 2015). The pH at which the reaction was conducted, did not seem to influence the changes in fluorescence. The blank sample showed stable fluorescence values throughout the reaction while all other samples showed a fast decrease, with a drop of roughly 70% during the first 30 min followed by a slowed-down further decrease of 10% till the end of the reaction. Resulting in an overall 80% decrease in fluorescence throughout the entire reaction time. Further, the addition of fluorophenol did not affect fluorescence. This was confirmed by the blank sample showing a stable fluorescence throughout the reaction and further by the POLY+FP sample where fluorescence did not change upon addition of FP after 150 min (Figure 3). Fluorescence is an intrinsic property of lignin, which is caused by conjugated phenylcoumarin, carbonyl, stilbene and biphenyl groups (Albinsson et al., 1999). Laccase oxidation of lignin leads to an electron transfer from phenolic hydrogen to molecular oxygen thereby, initiating the formation of phenoxy radicals on LS (Solomon et al., 2008). These radicals lead to a resonance stabilization in the aromatic ring system, subsequently disturbing the aromatic character, thus leading to the observed decrease in fluorescence potentially indicating coupling reactions (Albinsson et al., 1999).

In the course of this prescreening, it turned out that in terms of reactivity and increase in hydrophobicity the best results were achieved when the reaction was conducted at pH 7 (data not shown). These findings can be explained by the before mentioned interplay of reactivities between the substrates and the enzyme. In general, fungal laccases are known to have their optimal activity in a slightly acidic pH. Thus, one would expect the best results at pH 5. However, at this pH the structure of the LS is known to be more dense and compact, thereby hindering the steric interaction of the enzyme with LS. While at pH 7 the structure of LS is more open and thus shows higher reactivity while the enzyme should show lower activity. However, the herein used MtL is known to be an alkaline laccase, being active also at higher pH, hence it also shows reactivity at pH 7. At pH 8 the reactivity of LS decreases, due to deprotonation and thus predominance of phenolate anions. First, this sounds like the reactivity should increase, but in fact, it hinders the interaction between enzyme and LS due to increased electrostatic repulsion and a further decrease of enzyme activity with increasing pH at the same time. Also, hydroxide anions are known to inhibit laccase activity at high pH (Xu, 1997; Morozova et al., 2007; Vernekar, 2014; Singh, 2016; Ruwoldt, 2020). Based on these findings all following experiments were carried out at pH 7.

Besides the pH, parameters like temperature and substrate concentration are also known to influence reactivity (Morozova et al., 2007; Madhavi and Lele, 2009). Thus, in the next step, the temperature of the reaction was set to 33°C and the effects of different concentrations of FP added were investigated. Higher temperatures are generally expected to increase reaction rates while in this case changes in viscosity and therefore transport phenomena may additionally play a role. Especially for the herein used MtL enzyme, higher temperatures are favorable since it originates from a thermophilic microorganism (Singh, 2016). Further, it was shown that for the laccase catalyzed polymerization of LS the best results were achieved at a temperature of 33°C and that enzymatic modification efficiency increases with higher temperatures (Taghizadeh and Mehrdad, 2006; Weiss et al., 2020).

**FIGURE 4:**
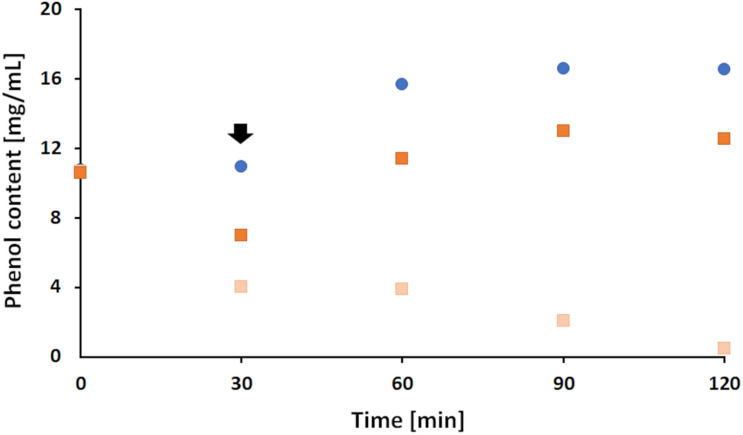
Changes in phenolic content during laccase catalyzed coupling of FP onto LS. Addition of 2% (w/v) FP after 30 min (indicated by the black arrow) to an ongoing polymerization of LS at 33°C and a pH of 7 (dark orange squares). The reaction was compared to a blank sample (blue dots) of LS and FP but without enzyme and to a control sample that contained LS and enzyme only (light orange squares).

The results showed that in the presence of laccase, a 48.1% decrease on average in phenol content was measured during the first 30 min while no change was observed for the blank sample containing LS only. However, upon addition of FP to the blank sample an expected increase of phenol content was seen remaining stable thereafter (Figure 4). This increase is linked to the structure of 4,4-F3MPP presenting a diphenyl ether, with a hydroxyl group on one aromatic ring and a functional CF_3_ group on the other (Figure 1C). Generally, the decrease in phenolic content during enzymatic oxidation of LS is attributed to the generation of LS reactive species leading to the formation of complex polymer structures, either among themselves or with other miscellaneous molecules (Areskogh et al., 2010). While the phenol content in the control sample (no FP) further decreased until the end of the reaction, upon addition of FP to the sample the phenol content increased by 46.3% in the modified sample and stayed stable afterwards (Figure 4). This indicated that the addition of FP might somehow prevent further polymerization. To further investigate this effect, changes in molecular weight and viscosity were determined in a next step.

**FIGURE 5:**
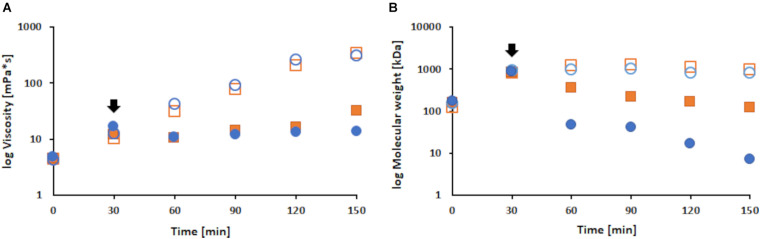
Changes in viscosity **(A)** and molecular weight **(B)** during laccase catalyzed coupling of FP onto LS at a pH of 7, 33°C and different concentrations of FP added. Shown are the 1% (w/v) FP sample (filled orange squares), the 2% (w/v) FP sample (filled blue dots) and the respective control samples (orange squares and blue dots). The black arrows indicate the addition of FP. To allow a better comparison the results are shown on a logarithmic scale.

Expectedly, the viscosity and molecular weight showed an increase during enzymatic polymerization of LS (Areskogh et al., 2010). In the absence of FP, due to extensive polymerization, the viscosity increased until the end of the reaction. However, the viscosity of FP modified samples decreased upon addition of FP, followed by a rather steady viscosity throughout the rest of the reaction. During the first 30 min of reaction, all samples showed a 2.9-fold increase in viscosity. While the control samples further increased viscosity up to 28.4-fold till the end, the addition of FP led to a decrease for the respective modified samples of 1.3-fold followed by a moderate increase of 2.2-fold (Figure 5A). The same trend was observed for the molecular weight results. During the first 30 min, all samples showed an increase of about 5.7-fold. Upon addition of FP, the 1% (w/v) sample decreased 2.3-fold and the 2% (w/v) FP sample 18-fold. Overall, the control samples increased molecular weight up to 6.5-fold, while the 1% (w/v) sample showed a 7-fold and the 2% (w/v) sample even a 123-fold decrease (examplary Chromatograms can be found in the Supplementary Material). The plateau that was reached for the control samples may suggest that no further polymerization occurs, or more likely that the increasing polymer size and hydrophobicity leads to an imprecise measurement (Figure 5B).

**FIGURE 6:**
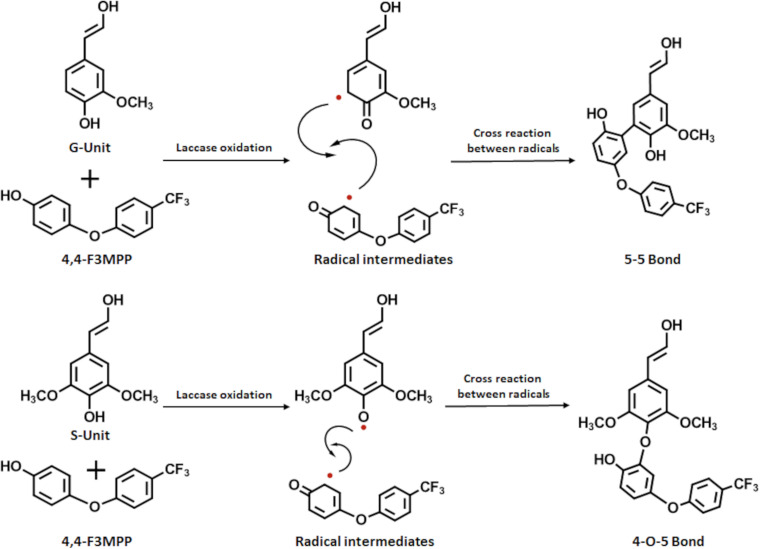
Scheme for the reaction between LS and FP modified from Kudanga et al. (2010). Laccase oxidation induces radical formation on both the LS and the FP leading to covalent bonds between the substrates. G units mainly form 5–5 bonds, while S units primarily form 4-O-5 bonds.

When laccase oxidizes LS it induces the formation of phenoxy radicals on the phenolic structures in LS. These radicals can cross-react among each other or with other phenolic structures thus, forming new linkages leading to an increase in polymer size. The observed decrease in molecular weight and viscosity in the presence of FP could be explained by incorporation of the added FP. The laccase does not only react with the LS but also with the FP molecules. The aromatic phenolic residue of the fluorophenol also forms phenoxy radicals which can cross-react with the LS radicals. This incorporation of fluorophenol could prevent further polymerization and even lead to depolymerization, resulting in subsequently smaller polymers (Fit̨igəu et al., 2015; Pezzella et al., 2015; Zavada et al., 2016). Investigation of the reaction mechanism for the incorporation of FP onto the forming LS polymers determined by NMR and MS measurements found that G residues primarily form 5–5 bonds, while S units mainly form 4-O-5 bonds (Figure 6; Kudanga et al., 2010).

Concerning the polymerization reaction, the results obtained so far showed, that extensive polymers were formed when the reaction was done at pH 7 and 33°C, which is in agreement with previously published data (Weiss et al., 2020). However, this study aimed to increase the hydrophobic properties of LS via enzymatic coupling of FP and not necessary extensive polymerization. To this end, hydrophobicity was monitored using the water contact angle (WCA) technique and the determination of changes in water absorption properties. In general, the contact angle between a surface and a drop of water deposited on it depends on the hydrophobicity of the surface. Materials showing a contact angle below 90° are defined as hydrophilic, whereas materials showing higher contact angles are hydrophobic (Bracco and Holst, 2013). Based on the observations made by Garcia-Ubasart et al. (2011) who stated that curing is needed to be able to detect the hydrophobic enhancing effect of FP, films were poured and cured after the reaction was finished and further used to determine the hydrophobic properties of the samples.

**FIGURE 7:**
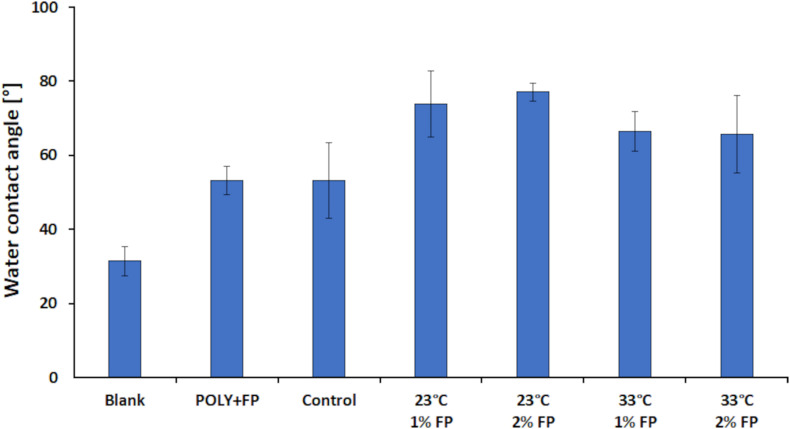
Results for the water contact angle measurement for the laccase catalyzed coupling of FP onto LS. All samples were done at pH 7. The blank was done without enzyme at 23°C and with 1% (w/v) FP added. In the POLY + FP sample, FP was added after the polymerization reaction was finished. The control sample was done with enzyme at 23°C and without FP added.

The WCA measurement showed a contact angle of 31.4° for the blank sample. While the control and POLY+FP samples showed an increase in contact angle to 53.1°. These results showed that enzymatically catalyzed polymerization of LS alone leads to an increase in hydrophobicity, due to the increase in insolubility with increasing polymer sizes. However, when both MtL and FP were added together to the reaction the contact angle increased further to 66° at 33°C and even to 75° at 23°C (Figure 7). Thus, for WCA measurement the best results were achieved when the reaction was carried out at 23°C and with 2% (w/v) FP added. These results further confirmed that both the enzyme and FP are needed simultaneously during the reaction for a successful enzymatic modification of LS.

Although none of the modified samples reached a contact angle above 90°, an overall increase of 59.2% in contact angle relative to the blank sample was achieved, representing a significant increase in hydrophobicity, which should be sufficient for better performance of LS treated in this way in coating formulations.

**FIGURE 8:**
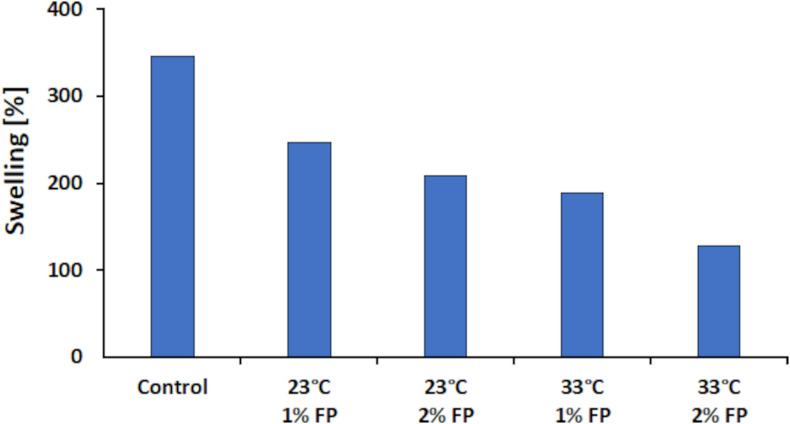
Swelling results for the laccase catalyzed coupling of FP onto LS. All reactions were conducted at pH 7. The control reaction was carried out at 23°C without FP added.

Besides WCA measurement, which allows determining hydrophobicity primarily on the surface of a sample, the swelling capacity allows for deeper insights on hydrophobicity. For this method, the amount of water absorbed by a material is gravimetrically determined and calculated according to the formulas (1) and (2) as presented in the section “Materials and Methods.” Other than the WCA measurement with this method the intermolecular hydrophobicity can be determined since water diffuses also into the inside of the sample. The more hydrophobic a sample is, the lower the swelling capacity should be (Thakur et al., 2017). Determination of swelling capacity was not possible for the blank sample, since it dissolved when immersed in water. This, however, evidenced that laccases are needed to convert water-soluble LS into water-insoluble polymers. Nevertheless, the control sample (laccase polymerized sample) showed a swelling capacity of 345.2%. In contrast, the FP modified samples showed a decreased swelling between 210 and 250% at 23°C and between 130 and 190% at 33°C. This represents a decrease in swelling of 220% between the control and the modified sample at 33°C with 2% (w/v) FP (Figure 8). The results showed that with higher concentrations of FP added, the lower the swelling was. When comparing the 1 to the 2% (w/v) FP sample at 23°C a 38.1% lower swelling was seen for the higher concentrated sample. This effect seemed to be more pronounced when the reaction was done at 33°C where the difference between the 1 and 2% (w/v) FP samples was 60% in swelling. Representing an overall decrease of 216.8% in swelling capacity for the 2% (w/v) FP sample at 33°C to the control sample at 23°C.

Overall, the best results concerning hydrophobicity were achieved when the reaction was carried out at pH 7 and 33°C with 2% (w/v) FP added. However, there is a small discrepancy when comparing the results for water contact angle and swelling capacity, since the WCA is higher for the samples done at 23°C than for those at 33°C. One possible explanation therefore may be the behavior of hydrophobic molecules in aqueous solutions. It is known that hydrophobic moieties, when brought into an aqueous milieu, tend to organize in a way that they point into the center of bigger molecules. The same was found for LS in aqueous solutions (Ruwoldt, 2020). While the WCA depends on the presence of hydrophobic molecules on the surface only, the swelling also depends on the internal structure of the polymers (Bracco and Holst, 2013; Thakur et al., 2017). Hence, it may be possible that most of the FP molecules were located primarily on the inside than on the surface. This may be the cause for the observed better results in swelling than in WCA measurement for the 33°C samples.

**FIGURE 9:**
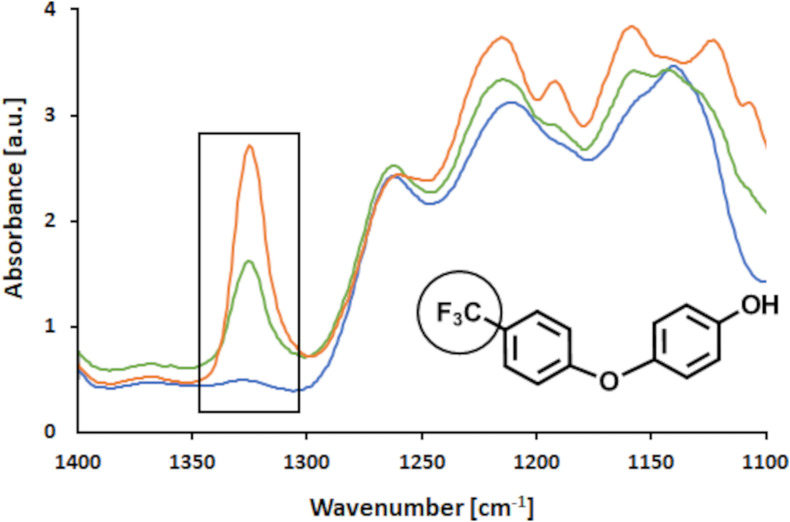
FTIR analysis of enzymatic coupling of FP to LS. FTIR spectrum in the range of 1,400–1,100 cm^– 1^, where the band of the –CF_3_ group is located. The control sample (blue), 1% (w/v) FP sample (green) and 2% (w/v) FP sample (orange) are shown.

Further, analyses of surface structural changes with FTIR after thorough washing with water and organic solvents showed that indeed the FP molecules had been covalently bound to LS in the presence of laccase (Slagman et al., 2018). As shown in Figure 9 the band for CF_3_ groups attached to an aromatic ring is located at 1,320 cm^–1^ in the FTIR spectrum (Socrates, 2001). Thus, only this region of interest of the spectrum between 1,100 and 1,400 cm^–1^ is presented here (the full spectra of the samples are presented in the Supplementary Material). It was observed that the control sample did not show a prominent band at 1,320 cm^–1^ whereas the modified samples did. Further, it was found that with higher concentrations of FP added the intensity of the band increased, suggesting that more of the CF_3_ groups were bound at higher concentrations added. When the concentration of added FP is doubled from 1 to 2% (w/v), a 1.7-fold intensity of the band was observed (Figure 9). However, it may be concluded that the addition of 2% (w/v) FP to the reaction, presented a too high concentration. Thus, due to restrictions in mass transfer, not all FP molecules added could have been incorporated into the LS polymer.

Further evidence of the successful covalent enzymatic coupling of FP onto LS was provided using XPS measurement. Comparison of the samples before and after washing showed that 2.45 at.% of FP indeed were detected at the end of the enzymatic coupling reaction. Whereas, washing led to a decrease to 1.89 at.% in detected Fluor atoms, suggesting that some adsorbed or only loosely bound FP molecules were removed upon washing (Table 1). However, the remaining FP molecules should be covalently bound onto LS. This is in agreement with our previous results, where covalent coupling of fluorophenols onto the complex lignin model compounds guaiacylglycerol β-guaiacyl ether and syringylglycerol β-guaiacyl ether was demonstrated by LC-MS and NMR analyses (Kudanga et al., 2010). Further analyses of the high-resolution C 1s spectra showed that enzymatic coupling led to a decrease in the relative content of C–O/C–OH bonds and further to the appearance of new C–F bonds. Expectedly, after the washing, the C–O/C–OH bonds showed an increase with a simultaneous decrease in Fluor content.

**TABLE 1:** Results of XPS analyses of the laccase catalyzed coupling of FP onto LS.

Sample	at.% C	STD	at.% O	STD	at.% F	STD	O/C ratio	F/C ratio
**Control**	72.4	0.9	23.8	0.9	–	–	0.33	–
**Control w^*a*^**	69.2	0.5	26.6	0.1	–	–	0.38	–
**Laccase 23°C 1% FP**	74.2	1.3	19.2	0.5	2.5	0.1	0.26	0.033
**Laccase 23°C 1% FP w^*a*^**	71.3	0.1	23.5	0.1	1.9	0.2	0.33	0.026

*The results are shown in atom percent (at.%). ^*a*^w, washed samples.*

## Conclusion

In this study, it was shown that it is possible to enzymatically modify isolated LS directly and thereby alter its properties by covalently coupling functional molecules. Here, it was the aim to increase the hydrophobicity of LS, which would be beneficial for its use in surface coating applications. The coupling of FP onto LS was found to be most efficient at pH 7 and 33°C leading to a significant increase in hydrophobicity, although a WCA above 90° was not reached. It was shown that the simultaneous presence of enzyme and functional molecules is a prerequisite for a successful coupling reaction. Both XPS and FTIR analysis indicated that covalent coupling of FP onto LS occurred. These studies show that it is indeed possible to tailor the properties of LS and produce functional hydrophobic coatings with potential applications in the wood and wood fiber industry. With the herein delivered proof of concept, it will be interesting in upcoming studies to test the reaction with different laccases, lignins and additives. Possible candidates to be used as additives are thought to be presented by lauryl gallate or unsaturated fatty acids, which were already shown to increase hydrophobicity when grafted on lignocelluloses (Garcia-Ubasart et al., 2011, 2012; Greimel et al., 2017). Another interesting approach would be the use of highly reactive lignin nanoparticles as a substrate (Legras-Lecarpentier et al., 2019; Chauhan, 2020).

## Data Availability Statement

The original contributions presented in the study are included in the article/Supplementary Material, further inquiries can be directed to the corresponding author.

## Author Contributions

All authors contributed to the writing of this manuscript and gave their approval for the final version.

## Conflict of Interest

NS and HW were employed by the company Sappi Papier Holding GmbH, Gratkorn, Austria. The remaining authors declare that the research was conducted in the absence of any commercial or financial relationships that could be construed as a potential conflict of interest.
