# Supplementary material for: Enzyme Catalyzed Copolymerization of Lignosulfonates for Hydrophobic Coatings
Source: Front Bioeng Biotechnol. 2021 Jul 14;9:697310. doi: 10.3389/fbioe.2021.697310 (PMC8317694; doi:10.3389/fbioe.2021.697310)
Supplement: Supplementary file 1 [file Table_1.DOCX]

Supplementary Material for

**Enzyme catalyzed copolymerization of lignosulfonates for hydrophobic coatings**

Sebastian A. Mayr^1^, Nikolaus Schwaiger^3^, Hedda K. Weber^3^, Janez Kovač^4^, Georg M. Guebitz^1, 2^, Gibson S. Nyanhongo^1,2^

^1^Institute of Environmental Biotechnology, University of Natural Resources and Life Sciences (BOKU), Konrad Lorenz Strasse 20, 3430 Tulln, Austria

^2^Austrian Centre for Industrial Biotechnology (ACIB), Konrad Lorenz Strasse 20, 3430 Tulln, Austria

^3^Sappi paper holding GmbH, Brucker Strasse 21, 8101 Gratkorn, Austria

^4^Jozef Stefan Institute, Jamova cesta 39, 1000 Ljubljana, Slovenia

Corresponding author:

Sebastian Mayr

[Sebastian.mayr@boku.ac.at](mailto:Sebastian.mayr@boku.ac.at)

**Full FTIR spectra of the samples:**


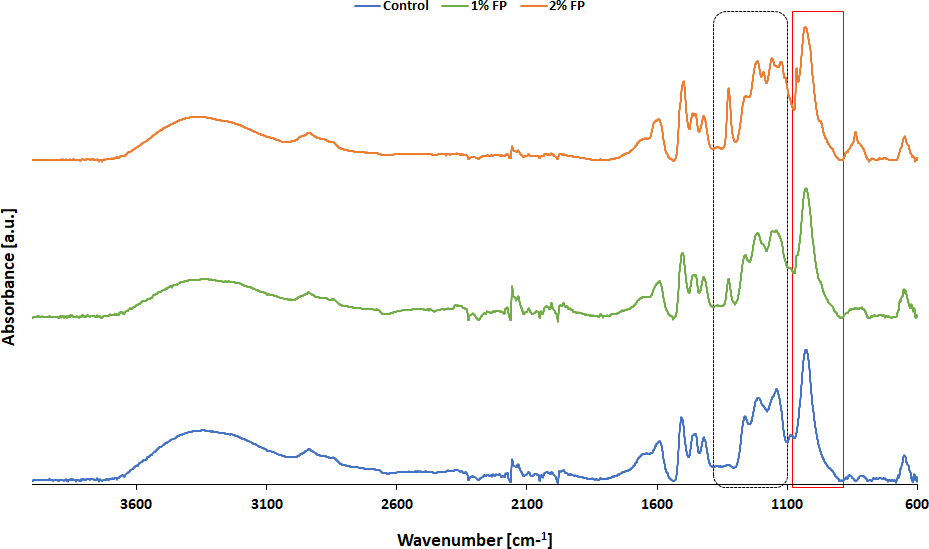


**Figure S1** Full FTIR spectra of the laccase catalyzed coupling of FP onto LS from 4000 to 600 cm^-1^. The black dashed box shows the zoomed in region from 1400 to 1100 cm^-1^ where the band for CF_3_ groups attached to an aromatic ring at 1320 cm^-1^ is located. The control sample (blue), 1% (w/v) FP (green) and 2% (w/v) FP sample (orange) are shown.The red square highlights the region where normalization was performed from 1070 to 930 cm^-1^ representing the fingerprint region of LS. Besides normalization the spectra were baseline corrected.

**Elution profiles of SEC measurements:**

**Figure S 2** Chromatogram of the 23°C control sample. The molecular weight increases throughout the reaction from TP 0 to TP 5.

**Figure S 3** Chromatogram of the 1% FP sample at 23°C. Molecular weight increases till TP 1, where FP was added, afterwards it showed a decrease, which may be linked to the incorporation of FP molecules.
